# P-136. Endocarditis Incidence Before and After Changes to Dental Prophylaxis Guidelines

**DOI:** 10.1093/ofid/ofaf695.363

**Published:** 2026-01-11

**Authors:** Michelle C Davidson, Kevin Ikuta

**Affiliations:** David Geffen School of Medicine at UCLA, Los Angeles, CA; West Los Angeles VA, Los Angeles, California

## Abstract

**Background:**

Infective endocarditis (IE) causes high morbidity and mortality worldwide, particularly in patients with underlying cardiac conditions. There is limited evidence on the effectiveness of antibiotic prophylaxis before dental procedures, and many cardiac societies have updated their guidelines to restrict prophylaxis to high-risk patients only, including the American Heart Association (AHA) in 2007, the European Society of Cardiology (ESC) in 2009, and other national societies. The aim of our study was to investigate whether the incidence of IE increased after guidelines became more restrictive.

**Figure 1.**
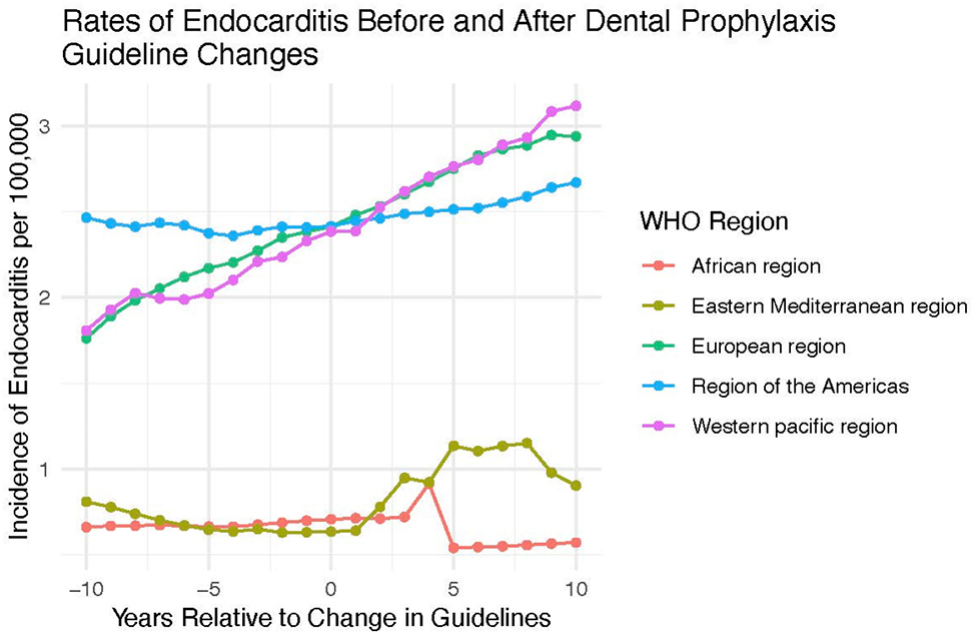
Rates of Endocarditis Before and After Dental Prophylaxis Guideline Changes

**Methods:**

We reviewed national guidelines for IE antibiotic prophylaxis through PubMed and country-specific cardiac society websites to identify countries that changed their guidelines to restrict prophylaxis to high-risk patients and the year this change occurred. We used the Global Burden of Disease Study 2021 to determine the incidence of IE for each country before and after their guidelines changed. Using an interrupted time series, we analyzed incidence rates of IE for the ten years before and ten years after each country’s change, grouped by WHO region.

**Results:**

We identified 66 countries with guidelines updated to restrict antibiotic prophylaxis to high-risk patients only. This included 54 ESC-member countries following ESC guidelines, three ESC member countries with their own guidelines, and nine non-ESC countries. Countries from each of the WHO regions were represented. Two countries had guidelines updated less than 10 years ago. Our interrupted time series analysis found a statistically significant increase in incidence rate of IE over time (p< 0.05); however, this change was not significantly associated with the timing of change in guidelines (p=0.90).

**Conclusion:**

Our findings suggest that IE incidence was not impacted by restricting antibiotic prophylaxis to high-risk patients only. This supports the current guidelines of the ESC, AHA, and other national guidelines with similar recommendations. Our findings support the need for additional research on the appropriate use of antibiotic prophylaxis for specific groups. Limitations of this study include the assumption that national guidelines were followed and that a limited number of countries had guidelines identified.

**Disclosures:**

All Authors: No reported disclosures

